# Effect of Perfluoroalkyl Endgroups on the Interactions of Tri-Block Copolymers with Monofluorinated F-DPPC Monolayers

**DOI:** 10.3390/polym9110555

**Published:** 2017-10-26

**Authors:** Syed W. H. Shah, Christian Schwieger, Zheng Li, Jörg Kressler, Alfred Blume

**Affiliations:** 1 Institute of Chemistry, Martin-Luther University Halle-Wittenberg, D 06099 Halle, Germany; syedwhshah@gmail.com (S.W.H.S.); christian.schwieger@chemie.uni-halle.de (C.S.); 18017742627@163.com (Z.L.); joerg.kressler@chemie.uni-halle.de (J.K.); 2 Chemistry Department, Hazara University, Mansehra 21120, Pakistan

**Keywords:** F-DPPC, polyphilic block copolymers, amphiphilic block copolymers, lipid monolayers

## Abstract

We studied the interaction of amphiphilic and triphilic polymers with monolayers prepared from F-DPPC (1-palmitoyl-2-(16-fluoropalmitoyl)-*sn*-glycero-3-phosphocholine), a phospholipid with a single fluorine atom at the terminus of the *sn*-2 chain, an analogue of dipalmitoyl-phosphatidylcholine (DPPC). The amphiphilic block copolymers contained a hydrophobic poly(propylene oxide) block flanked by hydrophilic poly(glycerol monomethacrylate) blocks (GP). F-GP was derived from GP by capping both termini with perfluoro-n-nonyl segments. We first studied the adsorption of GP and F-GP to lipid monolayers of F-DPPC. F-GP was inserted into the monolayer up to a surface pressure Π of 42.4 mN m^−1^, much higher than GP (32.5 mN m^−1^). We then studied isotherms of lipid-polymer mixtures co-spread at the air-water interface. With increasing polymer content in the mixture a continuous shift of the onset of the liquid-expanded (LE) to liquid-condensed (LC) transition towards higher molecular and higher area per lipid molecule was observed. F-GP had a larger effect than GP indicating that it needed more space. At a Π-value of 32 mN m^−1^, GP was excluded from the mixed monolayer, whereas F-GP stayed in F-DPPC monolayers up to 42 mN m^−1^. F-GP is thus more stably anchored in the monolayer up to higher surface pressures. Images of mixed monolayers were acquired using different fluorescent probes and showed the presence of perfluorinated segments of F-GP at LE-LC domain boundaries.

## 1. Introduction

Omega fluorinated fatty acids exist in nature. However, they are not widely distributed and their presence is limited to the *Dichapetalum* species [1]. No naturally occurring monofluorinated lipid has been found so far. Dipalmitoylphosphatidylcholine (DPPC) is one of the most employed lipids for mimicking biological membranes. The replacement of one hydrogen atom at the terminal methyl group of the *sn*-2 chain of DPPC by a single fluorine atom imparts peculiar properties to resulting lipid F-DPPC. In the beginning, the fluorine substitution in fatty acid chains of phospholipids was used only to probe the aggregation phenomena in membrane lipids [2]. But it was only after the revelation of interdigitation in the gel phase of F-DPPC by Hirsch [3] and coworkers that fueled the research on thermotropic phase behavior of this DPPC analog [4,5,6,7,8,9,10,11,12]. For lipid monolayers, however, only the effect of subphase temperature has been reported in pure F-DPPC and in mixed monolayers with DPPC [13]. Recently, we found in accordance with published data that not only this lipid forms an interdigitated gel phase [3], but also that the monolayer behavior is quite different from that of non-fluorinated DPPC. F-DPPC monolayers upon compression produce fractal 2D aggregation patterns, which differ from those of DPPC [14].

As the monolayer behavior of F-DPPC is quite different, we wondered, whether the interaction of block copolymers with and without fluorinated endcaps with these monolayers would also be different. The aggregation tendency of block copolymers in solution depends upon their architecture, and thus alters their potential for applications in industry and pharmacy [15,16,17,18,19]. Poloxamers, the triblock copolymers consisting of a poly(propylene oxide) block flanked by two poly(ethylene oxide) blocks are among those widely harnessed in biomimetic chemistry [20,21,22,23]. Though poloxamers were designated safe for use in cosmetics [24], a study indicated that they produced toxic degradation products on sonication [25]. The replacement of poly(ethylene oxide) block in these polymers by poly(glycerol monomethyl methacrylate) gives rise to a new type of polymers, which have greater ability to interact with membranes compared to poloxamers [18,26,27,28,29]. The polymer retention in membranes is further enhanced upon end capping of the amphiphilic triblock copolymers with perfluorinated moieties [30,31,32]. The resulting macromolecules are polyphilic, simply because their perfluorinated segments are hydrophobic as well as lipophobic [33]. The addition of fluorophilic component leads to peculiar characteristics [26,27,33,34,35]. For instance, hemifluorinated dibranch polymers produce stable fluorous emulsions, because they can prevent Oswald Ripening [36]. Also, the triphilic triblock copolymers produce micelles with defined fluorous regions [33,37].

**Figure 1:**
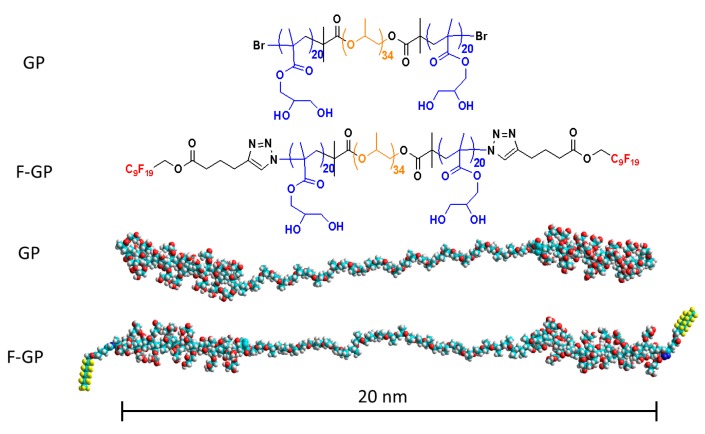
Chemical structures of amphiphilic block copolymer (GP) of poly(propylene oxide) (PPO) and poly(glycerol monomethacrylate) (PGMA) blocks, and triphilic block copolymer (F-GP) of PPO, PGMA, and F9 and their space-filling models.

There is plethora of information available on the role of block copolymers in biomimetic chemistry [38,39,40,41]. Fluorocarbons have exceptional properties that are highly relevant to medicine and pharmacy [42,43,44]. Efforts have been devoted to investigate their behavior in lipid membrane environment [45,46]. In order to understand the significance of polymers bearing such components, it is vital to study the interactions of these polymers with biological membranes or their simplified mimics [18]. The interactions between block copolymers and membrane lipids, including DPPC are affected upon α,ω perfluoroalkylation of block copolymers [31,32]. There is a solitary study available in literature on the effect of human serum albumin on F-DPPC monolayers [47], however the effect of polymers on monolayers or bilayers of F-DPPC has never been reported. In this article, we present data on the interaction with monolayers of monofluorinated F-DPPC with block copolymers with and without perfluorinated end caps. For this purpose, the adsorption of block copolymers to a pre-existing lipid monolayer of F-DPPC and their desorption from co-spread monolayers with the lipid have been studied. The tri-block amphiphilic copolymer, GP (PGMA_20_-PPO_34_-PGMA_20_) differs from its derivative, the triphilic polymer, FGP (F9-PGMA_20_-PPO_34_-PGMA_20_-F9) only in fluorocarbon segments (F9) attached to both sides. The structures of GP and F-GP are shown in Figure 1. The synthesis of these polymers has been reported elsewhere [29,48].

## 2. Materials and Methods

### 2.1. Materials

#### 2.1.1. Lipids and Lipid Probes

1-Palmitoyl-2-(16-fluoropalmitoyl)-*sn*-glycero-3-phosphocholine (F-DPPC) was purchased from Avanti Polar Lipids (Alabaster, AL, USA). Lipid probes 1,2-dipalmitoyl-*sn*-glycero-3-phospho ethanolamine-*N*-(lissamine rhodamine B sulfonyl)(triethylammonium salt) (RH-DHPE); 2-(12-(7-nitrobenz-2-oxa-1,3-diazol-4-yl)amino)dodecanoyl-1-hexadecanoyl-*sn*-glycero-3-phospho choline (NBD-12HPC) and 1,2-dipalmitoyl-*sn*-glycero-3-phosphoethanolamine-*N*-(7-nitro-2,1,3-benzosadiazol-4-yl)(triethylammonium salt) (NBD-DPPE) were from Invitrogen Karlsruhe, Germany. The purity of all these products was above 99% and they were used as received.

#### 2.1.2. Block Copolymers 

The synthesis and characterization of block copolymers, PGMA_20_-PPO_34_-PGMA_20_ (GP) and F9-PGMA_20_-PPO_34_-PGMA_20_-F9 (F-GP) has been reported elsewhere [29,48,49]. Briefly, the polymers were synthesized by a combination of ATRP with “click chemistry” for the attachment of the terminal moiety with the C_9_F_19_ group. The molar masses of GP and F-GP were 8555 g mol^−1^ and 9830 g mol^−1^, respectively. Both polymers had a polydispersity of 1.2.

#### 2.1.3. Other Chemicals

Chloroform and methanol of HPLC (high-performance liquid chromatography)-grade were products of Carl Roth GmbH & Co KG (Karlsruhe, Germany). Ultrapure water from a Milli-Q Advantage A10 System (Millipore S.A.S., Molsheim Cédex, France) was used as subphase and for the preparation of the solutions. The conductivity of water was below 0.055 μS cm^−1^ and the *TOC* (total organic carbon) value less than 5 ppb at 25 °C. The cleaning agent Hellmanex^®^ was obtained from Hellma GmbH (Müllheim, Germany).

### 2.2. Methods

#### 2.2.1. Pressure-Area Isotherms 

Pressure-area isotherms were recorded on a film balance having a trough with a maximal area of 536 cm^2^, two moveable barriers and a Wilhelmy plate (Riegler and Kirstein GmbH, Berlin, Germany). The trough was thoroughly rinsed using Hellmanex^®^ and deionized water. The pressure sensor was calibrated using the surface tensions of water and air. The uncertainties for the molecular area and surface pressure are usually ±2 Å^2^ molecule^−1^ and ±0.5 mN m^−1^, respectively.

Pure lipids, polymers, or their mixtures in desired ratios were spread at air-water interface from chloroform or chloroform/methanol (9:1) solution using a microsyringe from Hamilton (Bonaduz AG, Bonaduz, Switzerland). After allowing 15–30 min for evaporation of solvent and equilibration, the monolayer was compressed at a rate of 2.0 Å^2^ per lipid molecule per minute. An external circulating water bath (Haake Thermostat F3, Karlsruhe, Germany) was used to keep the temperature of subphase at 20 ± 0.1 °C.

#### 2.2.2. Time-Dependent Polymer Adsorption

The polymer adsorption from subphase to preformed lipid monolayers prepared at specific initial surface pressures was studied by using a system based on two homemade circular troughs (each with a diameter of 6 cm and depth of 0.3 cm) enclosed in a plastic hood. The surface pressure in each trough was monitored with a Wilhelmy plate (Riegler and Kirstein GmbH, Berlin, Germany). Before an experimental run troughs were thoroughly cleaned with Hellmanex^®^ and deionized water, and calibrated using the surface tensions of water and air. The sub-phase was kept at 20 ± 0.1 °C using an external water thermostat (Haake Thermostat F3, Karlsruhe, Germany). A defined volume of a lipid solution in chloroform was spread onto the water surface using a microsyringe from Hamilton (Bonaduz AG, Bonaduz, Switzerland) to achieve the desired initial surface pressure. When the surface pressure became constant, a fixed volume of the polymer solution was injected underneath the lipid monolayer to achieve a polymer concentration of 100 or 200 nM in the subphase. The subphase was gently stirred using a small spherical magnetic stirrer to enhance diffusion of the polymers to the surface.

#### 2.2.3. Epifluorescence Microscopy

Monolayers were visualized using an Axio Scope A1 Vario epifluorescence microscope from Carl Zeiss Microimaging (Jena, Germany). The microscope was fitted with a highly sensitive EMCCD (electron-multiplying CCD )camera (ImageEM C9100-13, Hamamatsu, Germany). A film balance comprising of a trough with moveable barriers and a Wilhelmy plate (Riegler and Kirstein GmbH, Berlin, Germany) was used. The maximal area of the trough was 264 cm^2^. The system was placed in a Plexiglas chamber and mounted on a moveable stage (Märzhäuser, Wetzlar, Germany). The recordings were done at 20 °C. The temperature fluctuations were maintained at ±0.1 °C using a Haake Thermostat F3 (Karlsruhe, Germany). To a lipid in chloroform or lipid/polymer mixture in 9:1 chloroform/methanol solution the desired amount of fluorescently labeled lipid (0.01 mol % RH-DHPE or 1.0 mol % NBD-12HPC or NBD-DPPE) was added and a defined volume of the resulting solution was spread at the air-water interface using a micro syringe from Hamilton Bonaduz AG (Bonaduz, Switzerland). After solvent evaporation and equilibration, the monolayer was compressed at the rate of 2.0 Å^2^ per minute per molecule. The imaging of monolayer was continued throughout the experiment and images were acquired at different areas and surface pressures.

## 3. Results and Discussion

### 3.1. Surface Behavior of Pure Polymers and Pure F-DPPC at the Air/Water Interface

**Figure 2:**
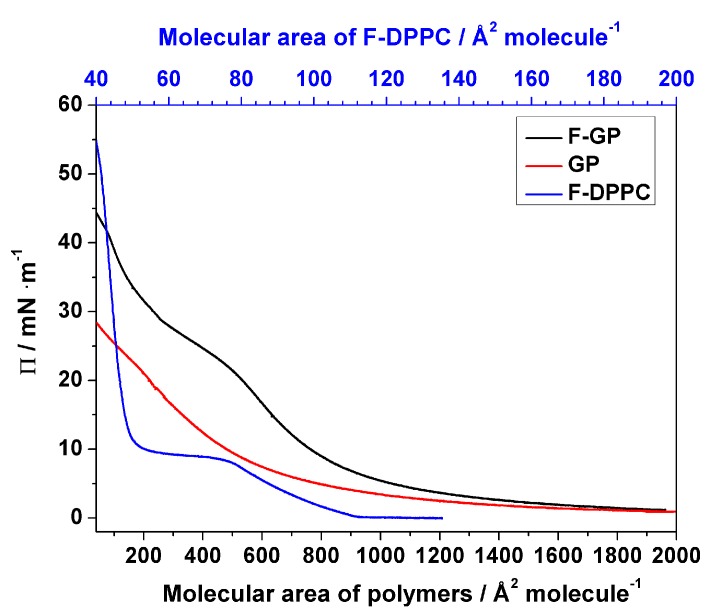
Compression isotherms for F-DPPC and the polymers GP and F-GP on aqueous subphase at 20 °C.

A detailed analysis of the surface behavior of GP and F-GP has been published recently by our group [32]. Figure 2 shows the compression isotherms of the pure polymers and pure F-DPPC after spreading at the air-water interface. In short, the block copolymers remain in a pan-cake regime when spread at the air/water interface and the available surface area is enough to accommodate the whole length of the macromolecules. With decrease in available surface upon compression, a shift of conformation to mushroom or brush takes place, where hydrophilic PGMA blocks are extended into the sub-phase. The polymeric monolayers are still compressible at this stage and the exclusion or retention of macromolecules is dependent on perfluoroalkyl segments. The perfluoro-n-nonyl chains make F-GP more surface-active compared to GP. This behavior was confirmed in this study. For adsorption experiments the polymers were injected into the aqueous subphase and the resulting surface pressure was recorded as a function of time. When the sub-phase concentration of polymers was 200 nM, their adsorption to the air-water interface lead to an equilibrium surface pressure of 13 and 24 mN m^−1^ for GP and F-GP, respectively. This clearly indicates that perfluoroalkylation makes the polymer more surface-active. F-DPPC has a much smaller area requirement than the polymers and the lift-of pressure is reached at 110 Å^2^ molecule^−1^.

### 3.2. Time-Dependent Adsorption of Polymers to Preformed Lipid Monolayers

It has already been established that addition of fluorinated segments to an amphiphilic polymer results in an increased affinity for lipid monolayers and bilayers [31]. This trait emerges from the hydrophobicity of perfluoro-n-nonyl chains [32], which provides a driving force for incorporation into the lipid alkyl chain regions, despite the fact that fluorocarbons do not mix with normal alkanes. In accordance with these observations, molecular dynamics (MD) simulations performed for perfluoroalkanes in a DPPC membrane showed self-aggregation of perfluoroalkanes F8 and F10 [46]. However, the aggregation tendency was reduced upon addition of a polar head-group, as in the case of perfluorinated *n*-alkanols and n-alkanoic acids [46].

In F-DPPC, the *sn*-2 chain terminus is polar and there is a possibility of back folding of the fatty acid chain as observed in lipids with a terminal fluorescent moiety, leading to a preferential localization of the C-F dipole at the air-water interface [50,51]. Hence, the conformation of the lipids in the LE phase of F-DPPC is different from that of DPPC, because in DPPC monolayers the alkyl chains avoid contact with water. In the LC phase at 30 mN m^−1^, both DPPC and F-DPPC exhibit comparable chain tilt from the surface normal indicating that the terminal –CH_2_F group is removed from the air-water interface upon compression [14].

To investigate the adsorption of polymers with and without fluorinated chains to F-DPPC monolayers, the polymers were injected underneath pre-existing lipid monolayers prepared at different initial surface pressures (Π_ini_). The total concentration of both polymers in the subphase was 200 nM, which was well below their critical aggregation concentration (cac), i.e., 1–2 µM [30,32]. In each case, an increase in surface pressure was observed after polymer injection indicating their adsorption to the surface. Though the polymer adsorption to the surface is mainly driven by the hydrophobic PPO block, an interaction of the hydroxyl groups of the PGMA blocks with the lipid head groups provides an additional driving force for polymer incorporation into lipid monolayers. The net change in surface pressure is reflective of both interactions mentioned above. When the lipid is in the LE phase, the surface is less crowded and macromolecules can easily reach the surface. Both, polymer and lipid contribute to the surface pressure at this stage. After the onset of the LE-LC transition, a certain percentage of lipid already exists in the LC phase, so that polymer adsorption is reduced. Therefore, with greater coverage of the surface area by the lipid, a decrease in ΔΠ (=Π_max_ − Π_ini_) is observed. At high initial surface pressure, the surface contains only densely packed lipids and the polymer is unable to reach the air-water surface. At this stage, ΔΠ falls to zero and the polymer is virtually excluded from the lipid phase. This initial surface pressure at which ΔΠ = 0 is called the maximum insertion pressure (MIP) or exclusion pressure (Π_e_).

**Figure 3:**
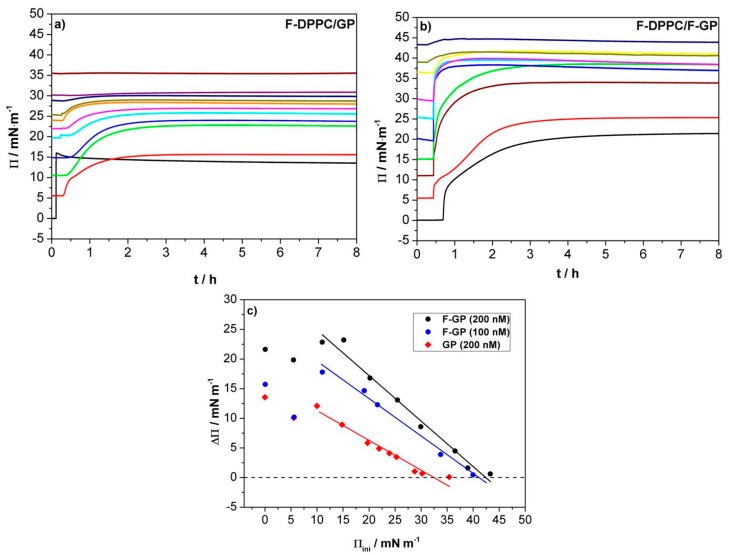
Time-dependent adsorption of polymers to F-DPPC monolayers spread at different initial pressures: (**a**) 200 nM GP; (**b**) 200 nM F-GP; and (**c**) Plots of ΔΠ vs. Π_ini_ for F-GP and GP. The blue circles show the adsorption of 100 nM F-GP from the subphase. The maximum insertion pressure (MIP) values for 200 nM GP and F-GP are 32.5 and 42.4 mN m^−1^, respectively.

For the case of GP that bears only PPO and PGMA blocks, the experimental curves are shown in Figure 3a,c. Similar trends are observed with triphilic F-GP, when injected underneath the preformed lipid monolayers (see Figure 3b,c). The overall behavior is similar to the observations reported before for adsorption to monolayers of normal DPPC [31]. However, there are some notable differences in the absolute values of ΔΠ when the GP and F-GP adsorption is compared, namely, ΔΠ_FGP_ is higher than ΔΠ_GP_ at the same Π_ini_ values. Compared to the adsorption to normal DPPC monolayers, there is also a markable difference. At an initial surface pressure of 5 mN m^−1^, the change in surface pressure caused by GP or F-GP binding to F-DPPC in the LE-phase is lower than that observed for the adsorption of the pure polymer to the bare water surface in contrast to the adsorption to DPPC monolayers [32]. This seems to be due to the fact that the *sn*-2 fatty acid chain of F-DPPC is back-folded and that the terminal CH_2_F group is located at the air-water interface. Adsorption of the polymers apparently leads to a detachment of the terminal group from the interface with a concomitant condensation of the monlayer, i.e., a drop in surface pressure. Consequently, ΔΠ is lower than for adsorption to DPPC in the LE-phase [32]. This effect vanishes when the preformed lipid monolayer is in the LC phase and the fluorinated chain terminus is already removed from the interface and pointing into the air parallel to the *sn*-1 chain. The maximum insertion pressure MIP for F-GP (100 nM) adsorption to a DPPC monolayer reported previously [32] and that observed in this study for F-DPPC monolayers (Figure 3c) are almost the same, namely 40–41 mN m^−1^. This endorses our finding that DPPC and F-DPPC behave very similar in the condensed LC phase [14]. When the polymer concentration of F-GP in the subphase is 200 nM, the MIP value increases only slightly to 42 mN m^−1^. The high MIP value for F-GP reflect an increased efficiency of the polymer with perfluoroalkylated end-caps to insert into membranes compared to GP.

Another marked difference between adsorption of GP and F-GP to F-DPPC monolayers is the kinetics of adsorption. It was observed before that F-GP adsorbs more slowly to the bare air-water surface and this was interpreted as being caused by more complex conformational changes of F-GP when reaching the surface [32]. When the kinetics of adsorption to DPPC and F-DPPC monolayers are compared it is quite evident that the kinetics has two different time regimes in case the F-DPPC monolayer is in the LE-phase. A fast process is followed by a slower one and this occurs for both polymers, GP as well as F-GP. This behavior disappears when the F-DPPC monolayer is in the LC-phase. The kinetic behavior for adsorption to LE-phase F-DPPC must therefore be caused by the special conformation of the *sn*-2 chain with the C-F group located at the air-water surface. It is likely that the first fast process is connected with adsorption of the first polymer molecules finding enough free space between the lipid molecules. For further adsorption of polymers, the terminal C-F group of the *sn*-2 chain has to be removed from the water surface leading to a slower adsorption process. The kinetics of the adsorption thus supports the explanation presented above for the peculiar decrease in ΔΠ for adsorption of polymers to LE-phase F-DPPC monolayers.

### 3.3. Compression Isotherms

**Figure 4:**
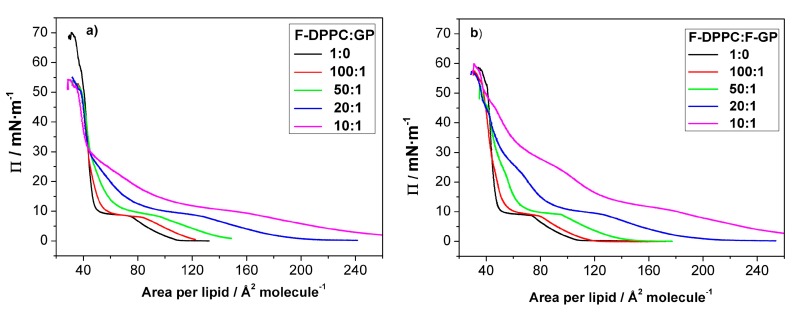
Compression isotherms for mixed lipid/polymer monolayers at the air-water interface: (**a**) F-DPPC/GP; and (**b**) F-DPPC/F-GP. The calculated molecular area was based on lipid content only.

We additionally studied a possible polymer desorption from co-spread lipid-polymer monolayers. To this end we first prepared mixed lipid-polymer films with different polymer content at the air-water interface and then compressed the mixed films until collapse occurred. The pressure-area isotherms of these mixed monolayers are shown in Figure 4. In each case, the LE-LC coexistence region is widened with respect to pure F-DPPC. The apparent area available to each lipid molecule increases with increasing proportion of polymer in the mixture as the polymer is taking up space at the interface (Table 1). The lipid phase change from gas to LE-phase is obscured due to an overlap of the pancake to mushroom transition in polymer arrangement at the interface [31].

In the prescence of the polymers the LE-LC transition occurs at a slightly higher lateral pressure as compared to the values observed for pure lipid monolayers. When the polymer content is low in the mixtures, the surface pressure is mainly determined by lipid. However, the lift-off area, i.e., the molecular area at which Π deviates from zero, is considerably higher due to the presence of the polymers. The value for the lift-off area increases with polymer content, indicating that more and more area is occupied by the polymer molecules. This increase in lift-off area is slightly higher when F-GP is present compared to GP. This shows again that F-GP molecules occupy more area at the interface than do GP molecules as already indicated by the isotherms of the pure polymers (see Figure 2). Upon compression, the surface pressure increases up to 8–10 mN m^−1^, where a kink in the isotherms, followed by a plateau, indicates that the lipid LE-LC transition is maintained in the presence of the polymers.

Above the LE to LC phase transition, the isotherms for F-DPPC/GP and F-DPPC/F-GP films are distinctly different. At the end of the phase-transition plateau, the area per lipid is still significantly higher when compared to the pure F-DPPC isotherms, indicating that the polymers are still located in between the lipid molecules of the film. The area occupied by the polymers is reduced upon further compression. In this pressure region, the isotherm is not as steep as the one for pure F-DPPC, i.e., an increased compressibility of the mixed monolayer is observed (Figure 4). Obviously, a gradual release of polymer molecules into the aqueous subphase occurs. Particularly, the isotherm for a 10:1 mixture with GP reveals a second “plateau” at a surface pressure of about 25 mN m^−1^. A similar plateau was observed before in films of DPPC with GP [32] and being interpreted as the beginning of the squeeze-out of the PPO blocks from the interface. For mixed monolayers of F-DPPC with GP, independent of the GP concentration, all isotherms coincide at about 30 mN m^−1^. At higher surface pressure, all isotherms have a steep slope, which is typical for pure LC-phase lipid monolayers. The area per lipid as well as the steep slope indicate that a complete squeeze-out of the polymer has now occurred.

The second “plateau”, where the PPO block is squeezed out of from the interface is more pronounced in F-DPPC monolayers with F-GP as compared to monolayers containing GP. At the end of the pseudo-plateau, the area is still significantly higher than that of the pure lipid, indicating that F-GP is still anchored at the interface via its fluorinated chains. The complete squeeze-out occurs at much higher pressure, i.e., at ca. 45 mN m^−1^. All these results indicate that F-GP is better anchored to the interface and less prone to squeeze-out than GP. The different squeeze-out pressure agree with the observations of the adsorption experiments shown above, where it was observed that F-GP has a higher MIP value of 42.4 mN m^−1^ compared to GP.

**Figure 5:**
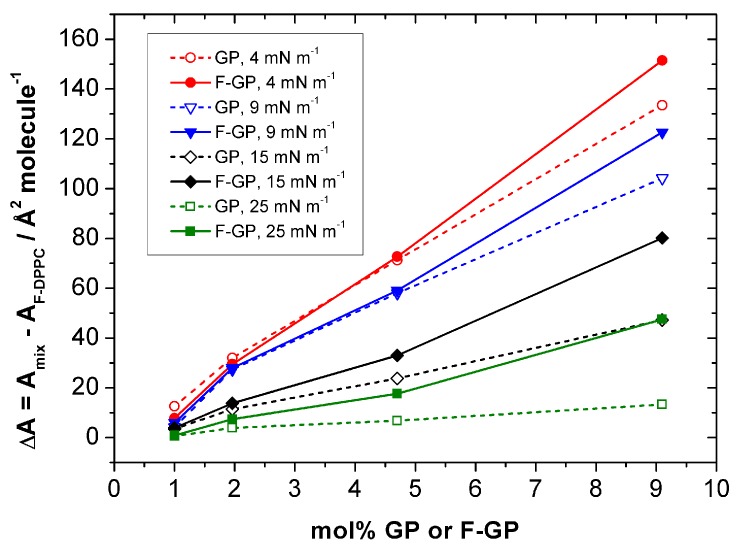
Shift in apparent molecular area per lipid molecule ΔA = A_mix_ − A_F-DPPC_ in a F-DPPC monolayer containing F-GP (filled symbols) and GP (open symbols) recorded at different surface pressures.

The differences between the two polymers in their affinity for F-DPPC monolayers can be quantified by comparing the changes in molecular area ΔA in the isotherms between pure F-DPPC and the isotherms of the mixtures at different surface pressure values, namely below the LE-LC transition in the LE-phase (4 mN m^−1^), at the onset of the LE-LC transition (9 mN m^−1^) and in the LC-phase below (15 mN m^−1^) and above the PPO squeeze out (25 mN m^−1^). A graph of these values is shown in Figure 5. It is quite evident from this comparison that F-GP is remaining in the film at higher pressure as the difference in ΔA-values for F-GP and GP become distinctly larger at higher surface pressure. Especially at 25 mN m^−1^ ΔA is close to zero for GP but still clearly positive for F-GP, indicating that GP is already released to the subphase whereas F-GP is still remaining at the interface at this surface pressure.

**Figure 6:**
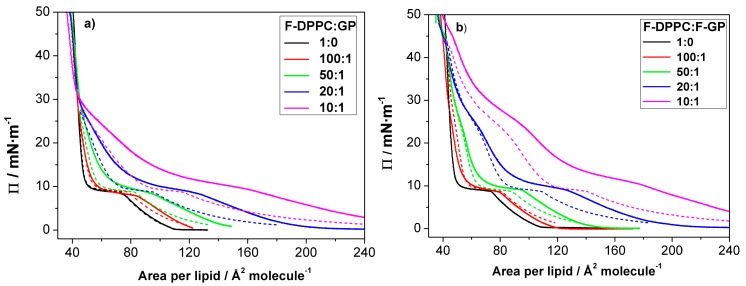
Experimental (full lines) and calculated (dashed lines) isotherms for mixed lipid/polymer monolayers at the air-water interface: (**a**) F-DPPC/GP; and (**b**) F-DPPC/F-GP. The calculated isotherms were obtained assuming additivity of the isotherms of the pure compounds scaled with their mole fractions in the mixtures.

Another question which arises is whether in co-spread lipid-polymer monolayers these two compounds mix in an ideal way, whether the polymers are in the same conformation in the mixed monolayer or whether some of the polymers are dissolving right after spreading into the subphase. These questions can be decided by calculating isotherms for the mixed films on the basis of the isotherms of the pure compounds by adding them scaled with their appropriate mole fraction in the mixed film according to the following equation:

$$A_{calc}\left(\Pi \right)=x_{polymer}A_{polymer}\left(\Pi \right)+x_{FDPPC}A_{FDPPC}\left(\Pi \right)\;$$

where $x_{\;}$ is the molar fraction of polymer or lipid in the mixed monolayer and *A* is the area per molecule in Å² at a given surface pressure. This yields calculated isotherms as expected for an ideal mixture or for total immiscibility, where the additivity rule also holds. Figure 6 shows calculated and experimental isotherms for the mixed films with GP and F-GP.

**Figure 7:**
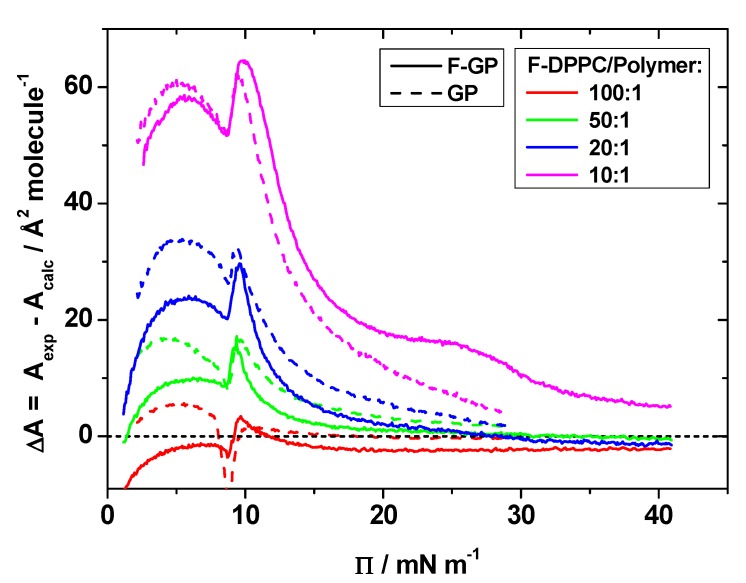
Difference in molecular area (ΔA) between experimental and calculated molecular areas as a function of surface pressure for mixtures of F-DPPC with GP and F-GP.

Figure 6 shows that the calculated isotherms are in most cases left-shifted to smaller molecular area, i.e., the experimental isotherms display larger area values at the same surface pressure. This is particularly evident for mixtures with a high polymer content. The differences become smaller with higher surface pressure, particularly for mixtures with GP, whereas they persist even at a surface pressure of 35 mN m^−1^ for the 10:1 mixture with F-GP. These results show that the additivity rule does not hold, meaning that the the mixtures behave neither ideally, nor are they totally demixed. Rather the mixture occupies more area than its two components in their neat phases. The differences between the effect of the two polymers become more evident when the differences in area between experimental and calculated molecular area are calculated. These are shown in Figure 7. It can be seen that the positive area excess of mixing is in general higher for GP than for F-GP. In other words: the mixing of F-DPPC and F-GP tends to be a bit more ideal than the mixing with GP.

The higher molecular area observed in the experiments can have different reasons: (1) in films of pure polymers spread at the air-water interface, more polymer is pushed into the subphase upon compression of the film than in lipid-polymer films. This would hint to additional attractive forces between lipid molecules and polymers in the film which keep the polymers anchored at the interface; (2) the polymer conformation in the mixed films is different compared to the conformation in the pure polymer monolayer. This could arise as a consequence of attractive interactions between the hydrophobic PPO blocks and the lipid chains keeping the hydrophobic block with its 34 PO (propylene oxide) units more flatly oriented at the air-water interface, thus needing more space than in the pure polymer film where the PPO block could also be oriented in a more random conformation at the interface; (3) the packing of the molecules at the interface is less ideal than in the neat phases, i.e., it is hampered by the prescence of the other component; and (4) all effects could be present at the same time.

The effects of GP and F-GP on F-DPPC monolayers can also be compared to their effect on DPPC-monolayers [32]. Several differences are apparent. Considering the pure F-DPPC monolayer, the transition pressure of a pure F-DPPC film at 20 °C is higher with ca. 9 mN m^−1^ than that in a pure DPPC-film (5 mN m^−1^). This difference is significant and is apparently caused by the necessity to remove the terminal fluorinated methyl group of the *sn*-2 chain from the water surface upon compression. Also, the lift-off area for F-DPPC is slightly higher with 110 Å^2^ molecule^−1^ compared to DPPC (90 Å^2^ molecule^−1^). Secondly, the the area-values at the onset of the LE-LC transition seem to be somewhat lower in F-DPPC than in DPPC films with incorporated F-GP polymers [32].

For mixed films with GP almost the same area values are obtained. For a 20:1 mixture, for instance, the area values at the onset of the LC-LE-transition are 130 Å^2^ molecule^−1^ for DPPC and 128 Å^2^ molecule^−1^ when the film contains F-DPPC. Also at other mixing ratios the area values in DPPC and F-DPPC monolayers with GP are almost the same. However, for incorporation of F-GP a pronounced difference in area is observed. For a 20:1 mixture with F-GP, the area values at the onset of the plateau region are 160 Å^2^ molecule^−1^ for a film with DPPC, whereas the value is 130 Å^2^ molecule^−1^ when the film contains F-DPPC. For a 50:1 mixture the difference persists. The area for films with DPPC is 115 Å^2^ molecule^−1^ whereas for F-DPPC films the area is again lower with 100 Å^2^ molecule^−1^. This means that the removal of the terminal fluorinated group of F-DPPC from the air-water surface upon compression on one hand occurs at a higher pressure independent of the nature of the polymer in the film, but the resulting mixed film is more condensed when F-GP is present in an F-DPPC film compared to a DPPC film. Part of this effect might be due to the higher transition pressure in F-DPPC but another reason for this difference might lie in the possible additional attractive interactions between the perfluorinated chain of F-GP and the terminal fluorine atoms in F-DPPC.

### 3.4. Epifluorescence Microscopy

The influence of the polymer on lipid phase change during compression was followed by epifluorescence microscopy. Two different dye labels were employed for this purpose: RH-DHPE, a dye which is excluded from the condensed lipid phase and NBD-DPPE, which partitions between LE and LC phases. These labeled lipid probes are shown in the Supporting Information (Figure S1). The microscopy images obtained for pure F-DPPC visualized using RH-DHPE were different from those of DPPC [33]. The usually bean-shaped domainswere distorted in the beginning of condensation, and later evolved to sea-weed like 2D aggregates. The images recorded with NBD-DPPE were bright white and attained flower like geometries upon compression (see Supplementary Figure S2).

**Figure 8:**
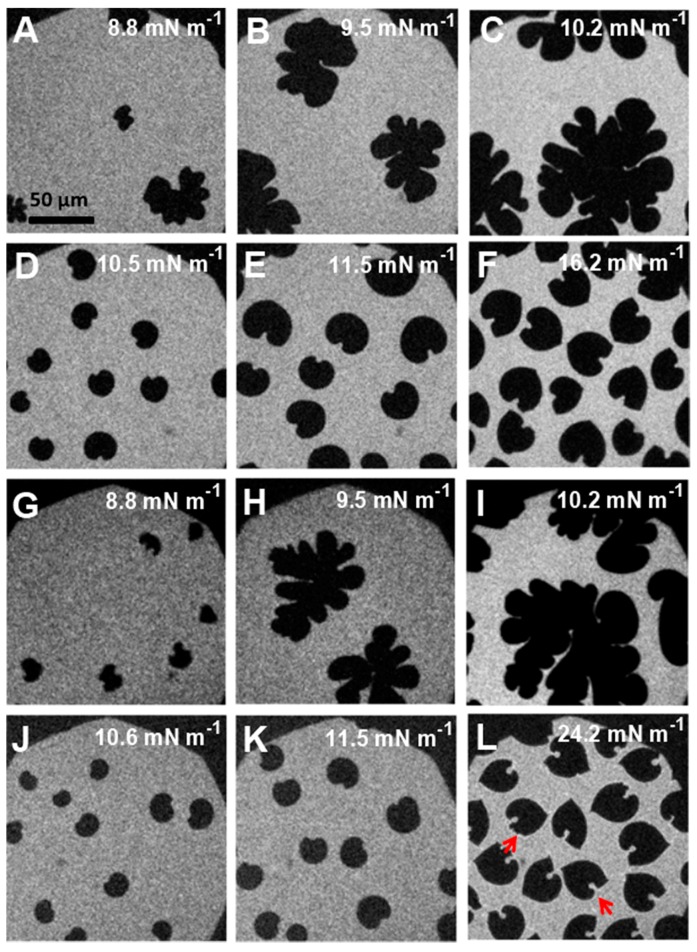
Epifluorescence microscopy images in mixed lipid/polymer monolayers recorded at different surface pressures: (**A**–**C**) F-DPPC/GP (50:1); (**D**–**F**) F-DPPC/GP (10:1); (**G**–**I**) F-DPPC/F-GP (50:1); and (**J**–**L**) F-DPPC/F-GP (10:1) co-spread monolayers containing 0.01% RH-DHPE. The corona around the domains in (**L**) is indicated by red arrows.

In mixed monolayers containing low amounts of polymers, the images were somewhat similar to those of pure F-DPPC. The domains were distorted and resembled extended cauliflower-like structures (Figure 8A–C,G–I). Effects produced by GP and F-GP on domain morphologies were very similar at low polymer content. An unexpected change in the domain morphology to bean or propeller shapes occurred for mixtures with higher polymer content (20:1 and 10:1) (Figure 8D–F,K–L). 

**Figure 9:**
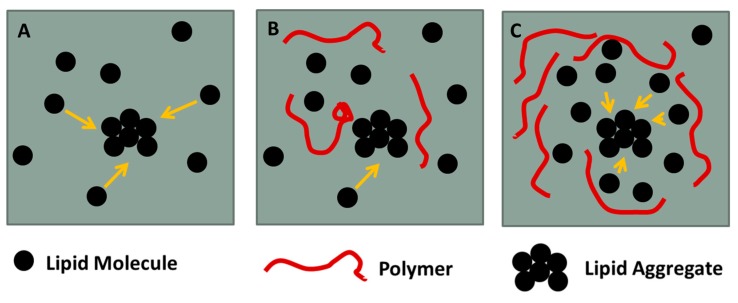
Lipid diffusion in (**A**) pure lipid; (**B**) hindered aggregation due to small polymer content; and (**C**) efficient lipid diffusion in lipid confinements. Yellow arrows mark the direction of the diffusion of the lipid molecules.

Small amounts of polymers apparently do not hinder fractal domain growth. However, after further polymer addition a significant part of surface area is occupied by macromolecules and lipid diffusion is hindered by the polymers present at the interface the stress produced by compression leads to adoption of different structural regimes of the polymer, such as pan-cake, mushroom and brush. The confined lipids in different islands act as nucleation sites for lipid condensation. This leads to a larger number of domains and to a domain growth upon further compression which is only possible by condensing lipids in the immediate vicinity of the domain boundaries due to the hindered diffusion. This is schematically shown in Figure 9. Thus the domains do not develop the typical fractal shapes as observed at low polymer content. The images recorded slightly above the end of the first plateau region (Π = 10–15 mN m^−1^) indicate clearly that essentially all of the lipids are in the LC phase. The remaining bright area where the lipid dye is located is occupied by the polymer. For instance for F-DPPC/GP films at a ratio of 10:1 and a surface pressure of 16.2 mN m^−1^ (Figure 8F), approximately 50% of the total area is covered by black domains. This corresponds to the area per lipid observed in the isotherm at this surface pressure, which is roughly twice as large as for the pure F-DPPC (see Figure 4a). For a mixture of F-DPPC with F-GP (10:1) this occurs at a higher pressure of ca. 24 mN m^−1^ (see Figure 8B,L). This is due to the fact that F-GP needs more space due to its fluorinated anchors.

A comparative analysis of FM-images obtained for 10:1 mixtures of F-DPPC with F-GP and GP revealed an additional feature of the polymer end-capped with F9 segments. A corona appeared around the condensed domain of an F-DPPC/F-GP film at Π ~ 24 mN m^−1^, indicating an accumulation of the rhodamine dye at the domain boundaries (Figure 8L). Since this feature is not seen in mixed films with GP, it can be inferred that the dye diffusion from domain edges to the bulk phase is hindered due the attached F9 segments to the polymer. It could be that the perfluorinated chains of F-GP are preferentially located at the boundaries of the condensed LC domains and prevent the diffusion of the lipid dye into the surrounding when it is expelled from the area where the condensed lipid domains form.

**Figure 10:**
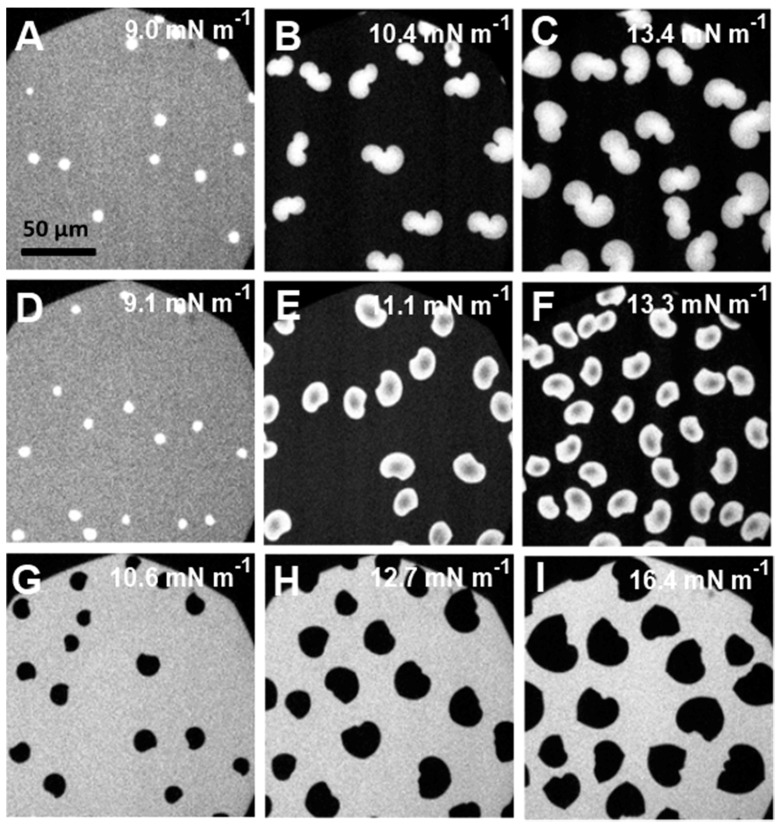
FM-images for F-DPPC/F-GP (10:1) co-spread monolayers recorded at different surface pressures in the presence of NBD labeled lipid probes: (**A**–**C**) NBD-DPPE on pure water subphase; (**D**–**F**) NBD-DPPE on 0.1 M aqueous NaCl subphase; and (**G**–**I**) NBD-12HPC on 0.1 M aqueous NaCl subphase. The label concentration in the mixture was 1.0 mol %.

We then used a different lipid probe, namely NBD-DPPE, which is known to partition into LC domains of the lipid monolayers due to its much smaller NBD moiety compared to the rhodamine residue. Images for 10:1 mixtures of F-DPPC with F-GP recorded with NBD-DPPE probe confirmed that the probe was accumulating in the LC lipid phase upon compression. These images are shown in Figure 10A–C. At low surface pressure, the LC-domains were small and round and had a higher fluorescent intensity as the surrounding LE-phase containing the polymer. However, the residual brightness of the LE-phase shows that NBD-DPPE also partitions into these areas. Upon compression, the LC-domains become brighter and peanut or propeller shaped. The background was increasingly darkened upon compression, showing dye accumulation in LC domains, i.e., NBD-DPPE does not partition any more in the areas outside the LC-domains, presumably because they contain only very little lipid as the LE-LC transition of F-DPPC is almost complete. A closer look at the LC domains reveals that the dye does not distribute evenly within the condensed lipid domains. At domain boundaries the intensity seems to be lower indicating some dye leakage into the areas containing mostly polymers.

Measurements were also carried out with films spread on a 0.1 M aqueous sodium chloride subphase to investigate the influence of ionic strength on the dye distribution as NBD-DPPE is a charged lipid dye, which has a negatively charged phosphate moiety (see Figure S1). At low surface pressure, the LC-domains were again round and had brighter intensity than the surrounding lipid LE phase containing the polymers (Figure 10D–F). Again, the background was darkened upon compression, indicating a reduced partitioning of the dye into the polymer-rich areas. When the film was spread on an aqueous salt solution the domain shapes were different in that they were more rounded. Also, the dye distribution in the domains was reversed, the centers being darker than the edges. Thus, the ionic strength of the subphase has an influence on the distribution of the dye. Due to the shielding of the negative charge at the phosphate moiety of the dye by the sodium ions of the subphase, the fluorescent molecules can probably more easily accumulate at the rims of the domains.

We also carried out similar experiment with a tail labeled probe, NBD-12HPC, which is molecule with a zwitterionic headgroup. The images are shown in Figure 10G–I. The distribution of this dye is similar to that of RH-DHPE and the observed domain shapes are consequently similar to those observed with RH-DHPE (see Figure 8J–L). The dye does not partition into the LC-domains of the lipid and stays in the polymer-rich phase.

## 4. Conclusions

We have studied the interaction of monofluorinated F-DPPC with triblock copolymers in monolayers at air-water interface. One of these polymers was amphiphilic, since it comprised only hydrophilic PGMA blocks and a hydrophobic PPO block. This polymer, designated as GP, was squeezed out of F-DPPC monolayers at a surface pressure of 32 mN m^−1^. The addition of perfluoro-*n*-nonyl segments at both ends of this polymer generated the polyphilic polymer F-GP. This led to polymer retention in the lipid monolayers up to 42 mN m^−1^. These results were very similar to those obtained with DPPC [32]. Different transitions involving polymer and lipid were visible in the pressure-area isotherms. Positive mixing excess areas showed that the mixing of the polymers with lipids is not ideal. Comparison of the behavior of F-GP mixed with DPPC or F-DPPC showed marked differences. Apparently, the additional attractive interaction between the single fluorinated methyl group of the lipid with the perfluorinated alkyl chains of the polymer leads to an enhanced condensation effect and to a more stable anchoring of F-GP in the F-DPPC monolayer when the monolayers are in the LE-phase or in the LE-LC transition region. The squeeze-out pressure for F-GP is only marginally higher for F-DPPC compared to DPPC. Epifluorescence microscopy imaging of the monolayer showed a dependence of domain formation on polymer content in mixed monolayers. The fractal growth of LC domains in mixed monolayers was augmented in 100:1 and 50:1 mixtures, whereas it was inhibited in 20:1 and 10:1 lipid-polymer mixtures. A corona at the domain boundaries reflected the hindered RH-DHPE diffusion into the surrounding polymer-rich phase, which is related to the presence of perfluorinated segments at the domain boundaries. This was confirmed by using NBD-DPPE as a probe which partitions between LC and LE lipid phases as long as lipid is present, but which upon compression is excluded from the polymer-rich phase. Our investigations on F-DPPC monolayers provided additional information on the possible role of ‘philicity’ of polymers with respect to membrane binding compared to that obtained by using non-fluorinated DPPC. The investigations showed that fluorination of the acyl chain termini of the lipids can enhance incorporation of fluorinated block copolymers into liquid-expanded monolayers and possibly also into liquid-crystalline bilayers.

## Figures and Tables

**Table 1:** Shift in apparent molecular area per lipid molecule (ΔA) in a monolayer after F-GP and GP addition recorded at different surface pressures.

Lipid/Polymer Ratio (Moles)	ΔA/Å^2^ Molecule^−1^ (F-DPPC) at Various Surface Pressures (Π)							
	4 mN m^−1^		9 mN m^−1^		15 mN m^−1^		25 mN m^−1^	
	F-DPPC/GP	F-DPPC/F-GP	F-DPPC/GP	F-DPPC/F-GP	F-DPPC/GP	F-DPPC/F-GP	F-DPPC/GP	F-DPPC/F-GP
100:1	12.6	7.8	4.2	5.9	3.5	3.9	0.6	0.9
50:1	32.0	29.5	27.3	28.0	11.4	13.8	3.9	7.4
20:1	71.3	72.7	58.0	59.1	23.8	33.0	6.8	17.6
10:1	133.5	151.5	104.2	122.6	47.2	80.2	13.3	47.5

## Supplementary Materials

The following are available online at www.mdpi.com/2073-4360/9/11/555/s1, Figure S1: Fluorescently labeled lipid probes, Figure S2: Fluorescence microscopy images of LC domains in F-DPPC monolayers.
