# Supplementary material for: Effect of Perfluoroalkyl Endgroups on the Interactions of Tri-Block Copolymers with Monofluorinated F-DPPC Monolayers
Source: Polymers (Basel). 2017 Oct 26;9(11):555. doi: 10.3390/polym9110555 (PMC6418721; doi:10.3390/polym9110555)
Supplement: Supplementary file 1 [file polymers-09-00555-s001.pdf]

Supporting Information

Effect of Perfluoroalkylation on the Interactions of Tri-block Copolymers with Monofluorinated DPPC Monolayers

Syed W. H. Shah[1,2], Christian Schwieger[1], Zheng Li[1], Jorg Kressler[1] and Alfred Blume[1,*]

[1] Institute of Chemistry, Martin-Luther University Halle-Wittenberg, D 06099, Halle (Saale), Germany
[2] Chemistry Department, Hazara University, Mansehra, Pakistan
* Correspondence: alfred.blume@chemie.uni-halle.de; Tel.: +49-345-55-25850

Content:

***Fluorescently Labeled Lipid Probes*---S1**

***Fluorescence Microscopy Images of LC domains in F-DPPC Monolayers*---------------------S2**

Fluorescently Labeled Lipid Probes

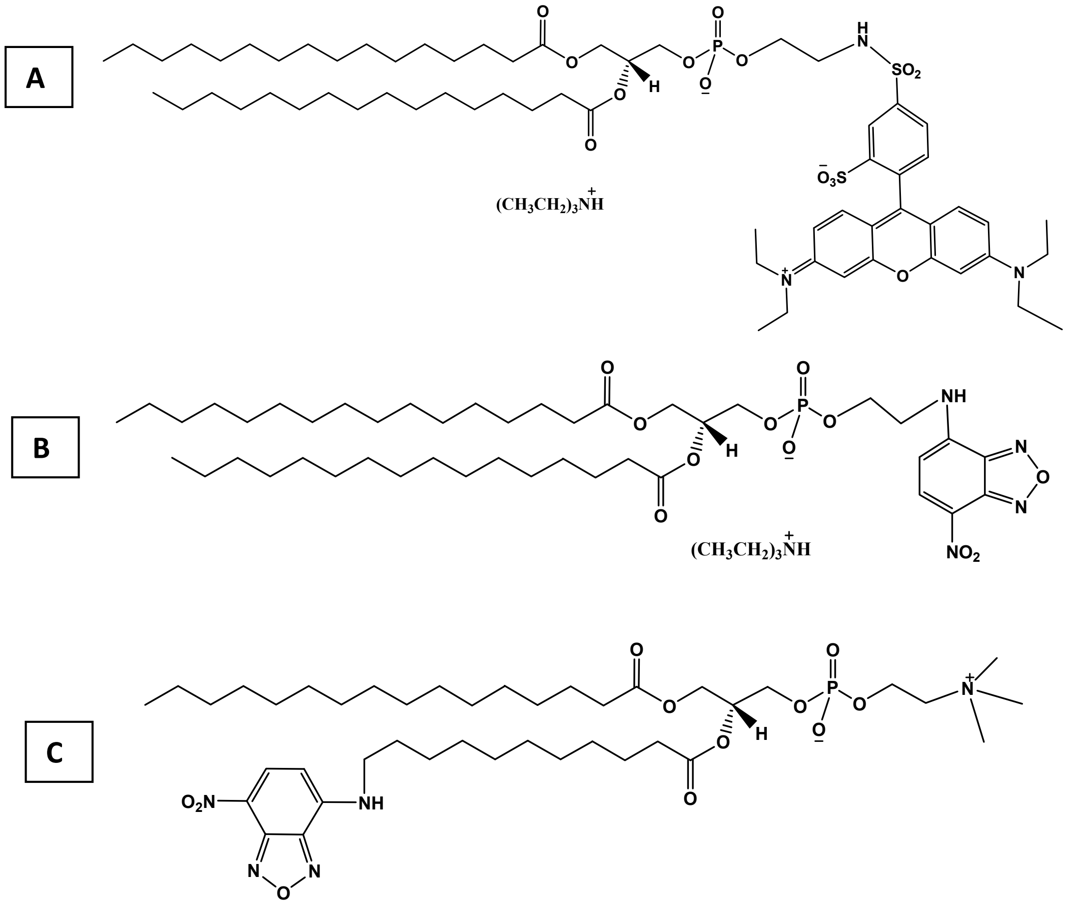

Figure S1. Chemical Structures of fluorescently labeled lipids: A) RH-DHPE; B) NBD-DPPE; and C) NBD-12HPC

Fluorescence Microscopy Images of LC domains in F-DPPC Monolayers

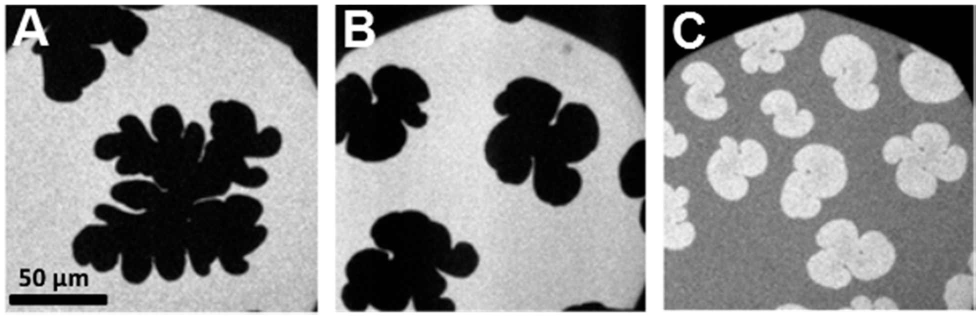

Figure S2. Epifluorescence microscopy images of F-DPPC monolayers in the presence of different labeled lipids recorded at 20°C: A) 9.9 mN m^{-1} with 0.01 mol% RH-DHPE; B) 9.4 mN m^{-1} with 1.0 mol% NBD-12HPC; and C) 9.3 mN m^{-1} with 1.0 mol% NBD-DPPE. The first two probes partition into the LE-domains, whereas NBD-DPPE partitions preferentially into the LC-domains, but is also present in the LE-domains, though with lower concentration. Therefore, the background in C is not completely black.
